# Mechanistic Insights into Inorganic Nitrite-Mediated Vasodilation of Isolated Aortic Rings under Oxidative/Hypertensive Conditions and S-Nitros(yl)ation of Proteins in Germ-Free Mice

**DOI:** 10.3390/biomedicines10030730

**Published:** 2022-03-21

**Authors:** Paul Stamm, Sanela Kalinovic, Matthias Oelze, Sebastian Steven, Alexander Czarnowski, Miroslava Kvandova, Franziska Bayer, Christoph Reinhardt, Thomas Münzel, Andreas Daiber

**Affiliations:** 1 Department of Cardiology, Cardiology I, University Medical Center Mainz, 55131 Mainz, Germany; paul.stamm@unimedizin-mainz.de (P.S.); sanela.kalinovic@unimedizin-mainz.de (S.K.); matthias.oelze@unimedizin-mainz.de (M.O.); sesteven@uni-mainz.de (S.S.); aczarnow@students.uni-mainz.de (A.C.); miroslava.kvandova@unimedizin-mainz.de (M.K.); 2 German Center for Cardiovascular Research (DZHK), Partner Site Rhine-Main, 55131 Mainz, Germany; christoph.reinhardt@unimedizin-mainz.de; 3 Center for Thrombosis and Hemostasis Mainz, University Medical Center Mainz, 55131 Mainz, Germany; franziska.bayer@kgu.de

**Keywords:** inorganic nitrite, oxidative stress, arterial hypertension, vascular function, metabolism and bioactivation

## Abstract

The prevalence and clinical importance of arterial hypertension are still growing. Inorganic nitrite (NO_2_^−^) represents an attractive dietary antihypertensive agent, but its metabolism and mode of action, which we aimed to investigate with the present study, are not completely understood. Isolated aortic rings from rats were treated ex vivo with oxidants, and rats were infused in vivo with angiotensin-II. Vascular responses to acetylcholine (ACh) and nitrite were assessed by isometric tension recording. The loss of vasodilatory potency in response to oxidants was much more pronounced for ACh as compared to nitrite ex vivo (but not in vivo with angiotensin-II). This effect may be caused by the redox regulation of conversion to xanthine oxidase (XO). Conventionally raised and germ-free mice were treated with nitrite by gavage, which did not improve ACh-mediated vasodilation, but did increase the plasma levels of S-nitros(yl)ated proteins in the conventionally-raised, but not in the germ-free mice. In conclusion, inorganic nitrite represents a dietary drug option to treat arterial hypertension in addition to already established pharmacological treatment. Short-term oxidative stress did not impair the vasodilatory properties of nitrite, which may be beneficial in cardiovascular disease patients. The gastrointestinal microbiome appears to play a key role in nitrite metabolism and bioactivation.

## 1. Introduction

Poorly controlled blood pressure is still the main risk factor for a premature death and loss of life quality [1,2,3]. Despite the increasing prevalence and thus the importance of arterial hypertension, less than 50% of patients show adequate blood pressure control due to a lack of effective pharmacotherapy and inadequate drug adherence [4,5,6]. Thus, the need to develop new drugs and treatment strategies [7] and, in particular, to increase patient compliance is undisputed [8].

For years, nitrite and nitrate intake have been accused of increasing the risk of cancer, since they are converted at least in part into carcinogenic nitrosamines, although this side effect is still under discussion [9,10]. In contrast, dietary nitrite increases the bioavailability of nitric oxide [11,12], which in turn may improve cardiovascular health, especially in patients with arterial hypertension [13,14]. The advantage of inorganic nitrite or nitrate therapy lies in part in its uncomplicated administration (e.g., consumption of nitrite/nitrate-rich vegetables) [15,16], which may increase therapy compliance significantly compared to the unpopular alternative of “pill-swallowing”.

Inorganic nitrite supplementation induces beneficial cardiovascular effects in hypertensive animals [17,18,19,20]. Additionally, Ling et al. showed, ex vivo in isolated aortic rings of rats, that sodium nitrite *per se* is a potent vasodilator and that its vasodilatory properties are not diminished in the aortic rings of spontaneously hypertensive rats [21]. To date, the reported effects of the gut microbiota on nitrite and nitrate bioactivation have been controversial, as shown by studies in germ-free mice (GF) or animals after antibiotic treatment [22,23,24]. The gut microbiota is a densely colonized microbial ecosystem, consisting of an assemblage of microorganisms, dominated by the bacterial genera Firmicutes and Bacteroidetes [25]. The microbiota has developed a mutualistic relationship with its host and therefore impacts many aspects of host metabolism, physiology and disease development [26,27]. Hence, animals are viewed as metaorganisms or holobionts, in which each partner fulfils a versatile and crucial role [28]. Although shotgun sequencing demonstrated remarkable differences between the human and mouse gut metagenome, there is a high similarity at the functional level, suggesting that gnotobiotic mouse models are suitable for studying microbiota–host interactions at the functional level [29]. The gut microbiota has emerged as a novel risk factor in cardiovascular disease, impacting the development of vascular dysfunction and hypertension [30,31,32]. Experimentation with germ-free mouse models has demonstrated that the vascular endothelium is strongly influenced by the presence of gut microbiota on the transcriptional level [33]. Since endothelium-dependent vasorelaxation is influenced by the gut microbiome [32,34], and the microbiota is involved in the bioactivation of nitrate [24], it is interesting to analyze the functional role of inorganic nitrite in vascular function. 

Zollbrecht et al. showed in 2016 that xanthine oxidase (XO) played an important role in nitrite reduction to nitric oxide (NO) in LPS-activated macrophages [35]. With the present study, we sought to determine the impact of different oxidants ex vivo on the vasodilatory potency of the endothelium-dependent vasodilator acetylcholine (ACh) compared to inorganic nitrite. We also sought to elucidate the impact of the gastrointestinal microbiome and of vascular xanthine oxidase levels on the nitrate bioactivation process.

## 2. Materials and Methods

### 2.1. Animal Treatment

All animals were treated in accordance with the Guide for the Care and Use of Laboratory Animals as adopted by the U.S. National Institutes of Health and approved by the Ethics Committee of the University Medical Center Mainz and the Landesuntersuchungsamt Rheinland-Pfalz (Koblenz, Germany; permit number: 23 177-07/G15-1-063 plus extension E1 and G18-1-001). In total, 48 male Wistar rats aged 6 weeks were used for harvesting aortic ring segments for vasodilation experiments and for an expression analysis of xanthine oxidase to dehydrogenase isoforms (Charles River, Sulzfeld, Germany). Angiotensin II infusion was performed for 7 days (AT-II, 1 mg/kg/d via Alzet osmotic pump model 2001, Cupertino, CA, USA) [36]. In total, 46 male mice with an age of 8–10 weeks were used. 32 of these were raised conventionally while 14 of them were born and raised under specific pathogen-free or germ-free (GF) conditions as previously described in [32] and adapted from [37]. Oral gavage with 0.9% saline (NaCl) or sodium nitrite (solution of NaNO_2_, single dose of 7.5 mg/kg) was performed in mice 2 h before organ removal in order to allow the distribution of nitrite through the body (as reported for nitrite and nitrate after oral administration in humans [38,39]) and sufficient conversion to nitric oxide to be monitored by protein-SNO formation (see Section 2.4). The aortic rings were used for isometric tension studies and measurement of the S-nitros(yl)ation of proteins in plasma. The animals were sacrificed by cardiac puncture upon isoflurane anesthesia and cervical dislocation. If not stated differentially, all chemical were from Sigma-Aldrich (Taufkirchen, Germany).

### 2.2. Isometric Tension Studies

After removing perivascular fat, the thoracic aorta was cut into 3–4 mm ring segments. For rat aortae, rings were then incubated separately for 90 min in a 100 mM potassium phosphate buffer with pH 7.4, at 37 °C, under continuous mixing, together with the respective oxidants (3-morpholinosydnonimine (Sin-1, Cayman Chemicals, Ann Arbor, MI, USA), H_2_O_2_, HOCl) at different concentrations. Isolated mouse aortic rings were not preincubated prior to the isometric tension recording. In organ bath chambers, isolated rings were connected to force transducers and vascular integrity was tested by potassium chloride-dependent vasoconstriction. Then, rings were preconstricted with phenylephrine for rats or prostaglandin F_2α_ (Cayman Chemicals) for mice, reaching roughly 70–80% of the maximal potassium chloride-induced tone. Subsequently, concentration–relaxation curves in response to increasing concentrations of ACh, NO_2_^−^ and nitroglycerin (GTN) were established as previously described in [36,40] and optimized from [41]. The acquisition system and software (Labchart version 8) used were manufactured by AD Instruments (Oxford, UK).

### 2.3. Western Blotting Analysis of Xanthine Oxidase and Dehydrogenase

The experiments were performed according to the manufacturer’s instructions and analogously to those already published [40]. Freshly incubated rat aortic rings were analyzed by Western blot analysis for xanthine oxidase and xanthine dehydrogenase (XO/XDH) using a rabbit monoclonal antibody (1:10,000, Rockland Immunochemicals, Pottstown, PA, USA). After incubation with a peroxidase-coupled secondary antibody (GAR-POX, 1:1000, Vector Laboratories, Burlingame, CA, USA) positive bands were detected by enhanced chemiluminescence. Visualization was performed using the ECL detection kit from Thermo Scientific, and Gel-Pro Analyzer 6.0 software (Media Cybernetics, Inc., Rockville, MD, USA) was used for densitometric quantification of the representative bands. 

### 2.4. Protein S-Nitros(yl)ation Quantification by Dot Blot Technique

Plasma samples were generated from venous blood obtained by heart puncture. 20 mM 5,5-dimethyl-1-pyrrolin-N-oxid (DMPO) was added and samples were illuminated with visible light with a wavelength of >420 nm for 30 min. Upon light irradiation, S-nitrosothiols underwent photolytic homolysis. The resulting thiyl radicals were converted to stable thionitrone products using DMPO [42]. Protein–DMPO adducts, representing the S-nitros(yl)ation of protein cysteine groups, were quantified by dot blot analysis using a DMPO-specific mouse monoclonal antibody (1:1000, Stress Marq Biosciences, Victoria, BC, Canada) according to the established protocols [40]. The secondary antibody was peroxidase-labeled (GAM-POX, 1:2000, Cell Signaling, Danvers, MA, USA). ECL development was performed as described above for the Western blot analysis. 

### 2.5. Statistical Analysis

Data are expressed as the mean ± standard error of the mean (SEM). Two-way ANOVA (with Bonferroni correction for comparison of multiple means) was used for comparisons of concentration–relaxation curves (Prism for Windows, version 9, GraphPad Software Inc., San Diego, CA, USA). One-way ANOVA (with Bonferroni correction for comparison of multiple means) was used for comparisons of XO/XDH as well as DMPO-positive protein levels (Prism for Windows, version 9, GraphPad Software Inc., CA, USA). All values passed a normality test (alpha = 0.05 in D’Agostino–Pearson tests, and Shapiro–Wilk tests for smaller numbers of values). We considered *p*-values < 0.05 as statistically significant. The number of measurements in the different assays may vary since not all animals were used in all assays.

## 3. Results

### 3.1. Effects of Different Oxidants on Endothelium-Dependent Vasodilation

**Figure 1:**
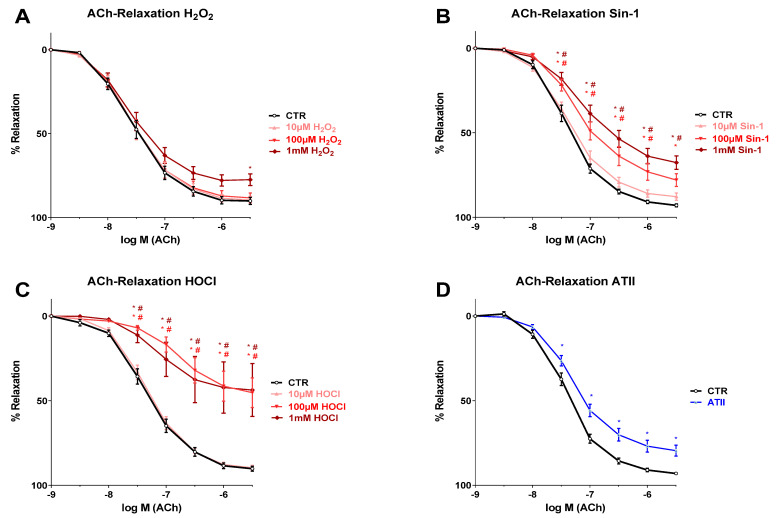
Effects of different oxidants on endothelium-dependent vasodilation. (**A**) Pre-incubation with 1 mM hydrogen peroxide (H_2_O_2_) caused endothelial dysfunction (impaired acetylcholine [ACh] response) while 10 µM and 100 µM H_2_O_2_ showed no relevant effect. (**B**) Endothelium-dependent relaxation showed a concentration-dependent deterioration when incubating with the peroxynitrite donor Sin-1. (**C**) Treatment with 100 µM and 1 mM hypochlorous acid (HOCl) impaired ACh-response, while the lowest concentration of HOCl showed no difference to untreated rings. (**D**) Endothelium-dependent vasodilation was impaired by ATII-infusion for 7 days. Data are presented as mean ± SEM from *n* = 14–16 (**A**,**B**,**D**), 7–14 (**C**) aortic rings/group. *p* < 0.05 * vs. Ctr; # vs. 10 µM.

Endothelium-dependent relaxation was measured by acetylcholine (ACh) response, which was impaired by pre-incubation with 1 mM hydrogen peroxide (H_2_O_2_), a clearly supra-physiological concentration of this oxidant, while lower concentrations (10 and 100 µM) of H_2_O_2_ caused no impairment (Figure 1A). When treating isolated aortic rings with the peroxynitrite donor Sin-1, a concentration-dependent deterioration of endothelium-dependent vasodilation that was significant at 100 µM and 1 mM Sin-1 was observed, whereas a marginal trend for impairment of the ACh-response was observed at 10 µM Sin-1 (Figure 1B). Since Sin-1 decomposes with a half-life of approximately 40 min under physiological conditions, the amounts used will generate steady-state-concentrations of peroxynitrite in the lower nanomolar range that can be considered physiological. The ACh-response was also clearly inhibited upon incubation with 100 µM and 1 mM hypochlorous acid (HOCl), which are again rather supra-physiological concentrations of this oxidant, whereas the low dose HOCl (10 µM) showed no effect at all (Figure 1C). The thoracic aortae of hypertensive (AT-II-infused) rats displayed a clearly impaired endothelium-dependent vasodilation (Figure 1D).

### 3.2. Effects of Different Oxidants on Nitrite-Induced Vasodilation

**Figure 2:**
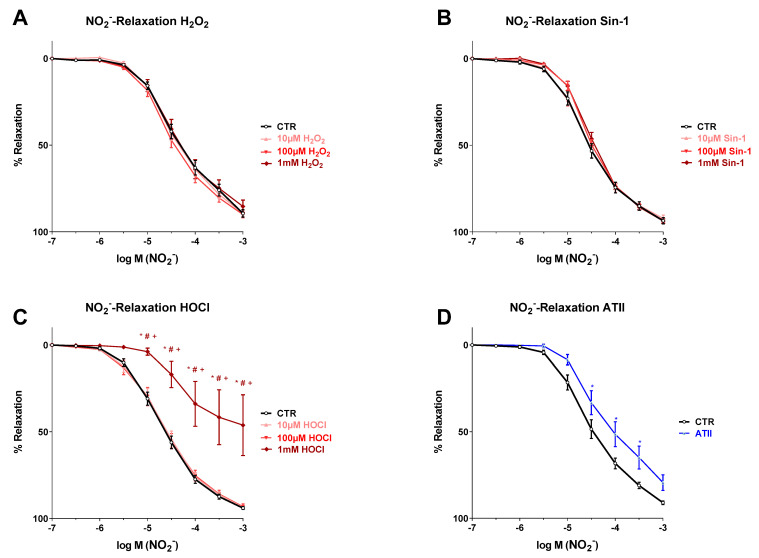
Effects of different oxidants on nitrite-dependent vasodilation. (**A**,**B**) All concentrations of hydrogen peroxide (H_2_O_2_) and the peroxynitrite donor Sin-1 had no effect on nitrite-induced vasodilation. (**C**) Nitrite-dependent aortic relaxation was not changed by low concentrations of hypochlorous acid (HOCl), while the highest concentration of HOCl (1 mM) clearly impaired vessel function. (**D**) Aortic rings of ATII-infused rats showed a worse response to nitrite than controls. Data are presented as mean ± SEM from *n* = 16 (**A**,**B**), 8–24 (**C**) and 15 (**D**) aortic rings/group. *p* < 0.05 * vs. Ctr; # vs. 10 µM; + vs. 100 µM.

All isolated aortic rings showed a clear and dose-dependent relaxation in response to cumulative nitrite concentrations. After pre-incubation with H_2_O_2_ or Sin-1, the nitrite-dependent relaxation was not impaired at all, not even at the high concentrations of 1 mM (Figure 2A,B). Upon incubation with HOCl at 10 and 100 µM, there was also no adverse effect at all on nitrite-dependent aortic relaxation, whereas pre-incubation with 1 mM HOCl led to a similar loss of vasodilatory potency of nitrite as observed for Ach-dependent vasodilation in the presence of higher HOCl concentrations (Figure 2C). Although the nitrite-dependent concentration–relaxation curve of the aortic rings of AT-II-infused rats showed a shift to the right (impairment) as compared to the controls (solvent only), the log-shift appeared to be less pronounced as compared to the Ach-response, which was also supported by the fact that the difference in nitrite-response between AT-II and control aortic rings was not significant at the highest nitrite concentration employed (Figure 2D).

### 3.3. Effects of Sin-1 on Xanthine Oxidase to Dehydrogenase Conversion in Aortic Rings

**Figure 3:**
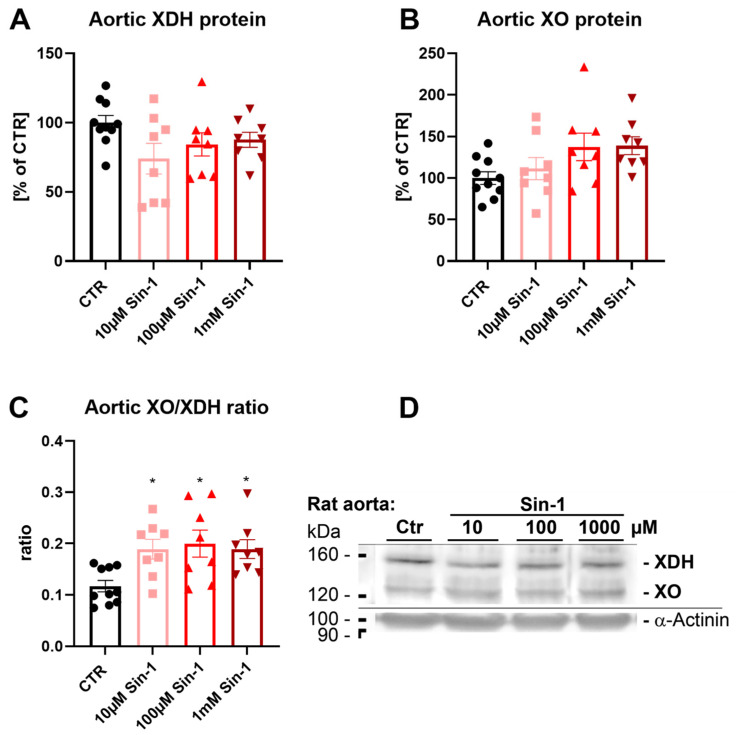
Effects of Sin-1 incubation at different concentrations on XO/XDH expression. (**A**) Levels of xanthine dehydrogenase (XDH) showed a decreasing trend by Sin-1 treatment. (**B**,**C**) The conversion to aortic xanthine oxidase (XO) protein form showed an increasing trend at higher concentrations of Sin-1, resulting in a significantly higher XO/XDH ratio in all Sin-1 treated aortic rings compared to the control group. (**D**) Representative original Western blots used for the quantification of protein expression displayed in Figure 3A,B. Data are presented as mean ± SEM from *n* = 8–10 (**A**–**D**) aortic rings/group. *p* < 0.05 * vs. Ctr.

Aortic xanthine dehydrogenase (XHD) protein levels were not changed significantly by different dosages of Sin-1 (Figure 3A). In contrast, there was an increased conversion to xanthine oxidase (XO) protein in response to incubation with higher concentrations of Sin-1 (100 µM and 1 mM) (Figure 3B). Consequently, the XO/XDH ratio was increased by Sin-1 incubation (Figure 3C), which may at least in part explain the failure of Sin-1 to impair the vasodilatory effects of inorganic nitrite (Figure 2B), e.g., by improved bioactivation of nitrite via XO.

### 3.4. Effects of Inorganic Nitrite Treatment on Vasodilation and S-Nitros(yl)ation

**Figure 4:**
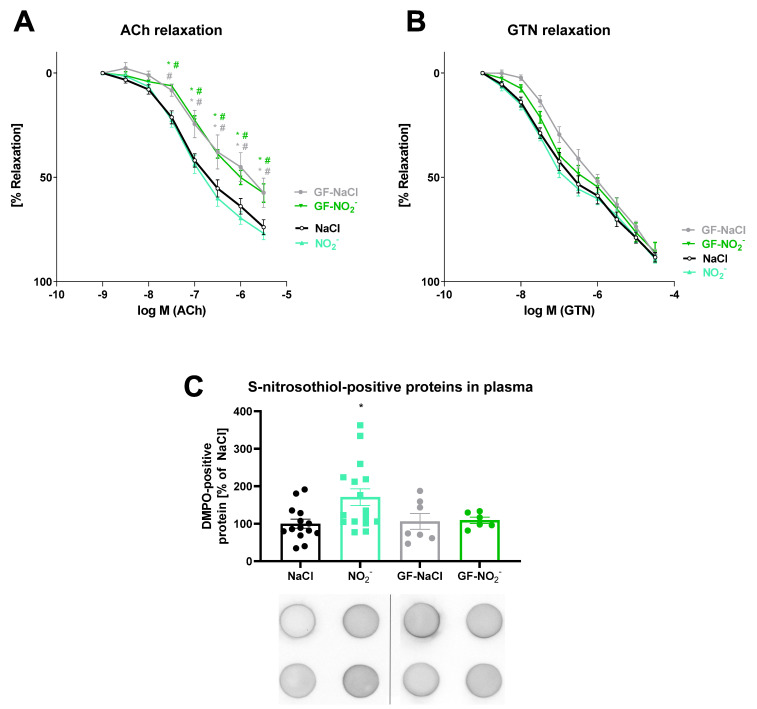
Effects of inorganic nitrite in vivo treatment on vasodilation and S-nitros(yl)ation of plasma proteins in the absence and presence of gut microbiota. (**A**,**B**) No changes in endothelium-dependent (ACh-response) and endothelium-independent (GTN-response) relaxation were observed after acute nitrite administration by gavage as compared with the solvent control (NaCl) in conventionally-raised (CONV-R) and germ-free (GF) mice. (**C**) Nitrite treatment resulted in increased plasma levels of S-nitrosothiol-positive proteins (by DMPO spin trapping); this effect was not present in GF animals. Data are presented as mean ± SEM from *n* = 6–14 (**A**,**B**) and 6–16 (**C**) animals/group. *p* < 0.05 * vs. NaCl; # vs. NO_2_^−^.

The endothelium-dependent (ACh) and endothelium-independent (GTN) vasodilation of isolated aortic rings was not changed by administration of inorganic nitrite via gavage as compared to the solvent control (NaCl) in conventionally-raised (CONV-R) and GF mice (Figure 4A,B). However, untreated conventionally-raised and GF animals differed in endothelium-dependent but not in endothelium-independent relaxation. DMPO-positive protein plasma levels, as a read-out for protein S-nitros(yl)ation, were increased by administration of nitrite via gavage in comparison with solvent (NaCl) treatment (Figure 4C). The increased formation of S-nitrosothiol-positive proteins in response to nitrite gavage was not detected in GF mice, indicating a lack of nitrite metabolism due to a missing gastrointestinal microbiome.

## 4. Discussion

In the present study, we sought to determine to what extent oxidative stress induced by various oxidants may impair nitrite-related, endothelium-independent vasodilation as compared to endothelium-dependent vasodilation in response to ACh in isolated aortic rings. In most instances, we observed an oxidant- and concentration-dependent impairment of endothelium-dependent dilation by ACh. In contrast, nitrite-induced vasodilation was much less sensitive to oxidants. For instance, only the highest concentration of HOCl (1 mM) significantly impaired nitrite-dependent relaxation. Thus, it seems likely that the oxidants may react with NO released by the eNOS after stimulation with ACh to form, e.g., the intermediate peroxynitrite [43,44,45], thereby reducing the potency of this endothelium-dependent vasodilator [46,47,48].

### 4.1. Mechanistic Considerations

The higher resistance of nitrite-dependent relaxation to oxidative stress may be best explained by the eNOS-independent nitrite bioactivation leading to large amounts of NO and a subsequent direct vasodilation of the smooth muscle [21,49]. A reduction of nitrite and a reduction of conversion to NO by heme proteins, such as ferrous hemoglobin or myoglobin, as well as the molybdo-enzyme xanthine oxidoreductase may induce sufficient activation of the soluble guanylyl cyclase in the smooth muscle, leading to relaxation [50,51,52]. In addition, a more efficient redox-dependent conversion of XDH to its oxidase form (XO) under oxidative stress conditions [53,54] will facilitate the bioactivation of nitrite to NO [35,55]. The higher resistance of nitrite-dependent vasodilation to oxidative stress may at least in part explain the overall beneficial profile observed for inorganic nitrite in various cardiovascular disease conditions that are associated with oxidative stress, such as experimental hypertension [17], diabetes/metabolic syndrome [56] and liver steatosis [57]. Likewise, the more efficient bioactivation of nitrite to nitric oxide in ischemic regions [58,59] may contribute to its overall beneficial cardiovascular profile [60,61].

In hypertensive rats (AT-II infusion), both the ACh-response and the nitrite-response were almost similarly impaired compared with the control group. However, there was a trend of less severe impairment of the nitrite-response as compared with the ACh-response, which is supported by previous observations [21]. These authors demonstrated an even stronger nitrite-induced vasodilation in aortic rings of spontaneously hypertensive rats (SHR) compared to corresponding vessels from control animals. 

The discrepancy with our results could be due to the induction of hypertension by treatment with the vasoconstrictor AT-II in our studies, whereas hypertension in SHR largely relies on remodelling by heritable mechanisms. There is evidence for corresponding differences with the therapeutic use of isosorbide-5-mononitrate (ISMN). ISMN improved the relaxation properties in SHR, whereas no difference was seen with simultaneous AT-II administration [36,62]. However, these differences between the hypertension models of SHR and AT-II infusion warrant further investigation. Within our study, a continuous therapy with AT-II in vivo appears to initiate processes damaging to the aorta other than those in the short term ex vivo incubation of isolated aortic rings with various oxidants, potentially involving oxidative damage of smooth muscle (e.g., at the level of the redox-sensitive soluble guanylyl cyclase [63,64]), all of which contributes to the impairment of nitrite-induced relaxation. It may be possible that, for example, peroxynitrite formation by Sin-1 does not reach the deeper smooth muscle cell layers when applied ex vivo, but that intrinsic formation of NO and superoxide radical by in vivo arterial hypertension generates enough intracellular peroxynitrite to cause the necessary oxidative damage in the smooth muscle cells that leads to impaired nitrite-response. This hypothesis is also supported by impaired GTN-dependent relaxation in aortic rings of hypertensive (AT-II infused) rats [36].

In order to investigate the acute effects of inorganic nitrite supplementation on the vascular function of the aorta in vivo, we chose oral gavage administration. In addition, to study the role of the commensal microbiota as a relevant modifier of the antihypertensive effect of inorganic nitrite, we investigated GF mouse models to gain additional metabolic insights. Two hours after the gavage with sodium nitrite (7.5 mg/kg), endothelial-dependent and endothelial-independent relaxation in aortic rings of CONV-R or GF mice were not modified in the nitrite-treated group compared to the respective solvent-treated control group. GF housing conditions per se lead to endothelial dysfunction, as indicated by an impairment of the dose–response relationship of the endothelium-dependent vasodilator ACh, while endothelium-independent NO-induced vasodilation with GTN was preserved. The lack of NO_2_-induced improvement of vascular function (no amelioration of ACh- or GTN-response) may be due to only one single administration and only a short-term (2 h) metabolism assessment, in contrast to previous reports, in which long-term treatment and metabolic effects were studied [65,66,67]. 

However, in our studies, even the short-term treatment and metabolism assessment showed a significant effect on S-nitrosocysteine-positive protein levels in the plasma of conventionally-raised but not of GF mice. Using a dot blot assay based on a specific antibody for DMPO-positive proteins (generated by DMPO spin trapping of protein thiyl radicals [42]), we were able to demonstrate that a single oral sodium nitrite uptake led to an instant increase in S-nitros(yl)ation of plasma proteins. During oral gavage, the applied substance only comes into contact with the oesophagus, the stomach and the distal gastrointestinal tract [68]. With these findings, we were able to confirm the previous study results showing that not only nitrate [23,24] but also nitrite metabolism is largely dependent on the gastrointestinal bacterial microbiome [69]. Thus, it is tempting to speculate that the changes in nitros(yl)ation observed in plasma did not yet reach the vascular system, explaining the lack of an effect on relaxation in our short-term and acute treatment experiment. However, our data on impaired endothelial function in GF mice (assessed by acetylcholine-dependent relaxation) are at variance with the reported rescue of endothelial function by broad-spectrum antibiotics treatment in mice fed a high-fat diet [34].

### 4.2. Clinical Considerations

Recent studies have shown a clear correlation between the gut microbiome and cardiovascular disease. Thus, the benefits of a Mediterranean diet seem to be mediated via the gut microbiome [70], and interactions between gut flora and arterial hypertension have been demonstrated [71]. A combined meta-analysis of all published human studies (with a total of 82 participants from 6 studies) on the impact of oral microbiota on plasma nitrite response by lowering blood pressure or increasing NOx species revealed a trend for a better response in subjects with intact oral microbiota (in groups without mouth washing) [72]. Since the intestinal flora is also strongly influenced by external circumstances, i.e., lifestyle, certain spurious correlations may come into play. It is of great importance to deepen the previous findings of a link between gastrointestinal and cardiovascular diseases and to thereby eliminate alleged confounders in further clinical studies. The finding that nitrite bioactivation also depends on the gastrointestinal microbiome, besides xanthine oxidase-dependent conversion, opens up several attractive therapeutic options, such as a dietary modulation of the gastrointestinal flora towards beneficial and healthy bacteria that may also be more efficient nitrite bioactivators. Probiotics in particular could help to promote inorganic nitrite bioactivation and thereby improve beneficial signalling by nitric oxide [73,74]. Both probiotics [75,76] and inorganic nitrite [77,78,79,80] have been shown to have multiple vasoprotective effects. Probiotics were also reported to activate the protective NRF-2 pathway and to increase eNOS expression in association with improved myocardial diastolic function in swine [81], and to prevent endothelial dysfunction in rats with portal hypertension [82]. However, larger clinical studies with a simultaneous application of both probiotics and inorganic nitrite are still lacking. Based on the present results, this symbiotic therapy should be used both pre-clinically and clinically, especially in cases of concomitant gastrointestinal and cardiovascular disease. Due to its low cost, simple properties and few side effects, this form of therapy could play an essential role in public health, especially in lower-income regions.

### 4.3. Limitations

One limitation of our study is that most experiments were performed ex vivo in isolated aortic ring segments, which may limit the relevance for the in vivo situation. Moreover, the investigated concentrations of oxidants (H_2_O_2_, HOCl) were rather supra-physiological, which limits the (patho)physiological conclusions regarding the impact of H_2_O_2_ and HOCl on endothelial function in comparison with nitrite-dependent vasodilation. Only the applied Sin-1 concentrations can be considered (patho)physiological, since Sin-1 decomposes with a half-life of approximately 40 min under physiological conditions, generating steady-state-concentrations of peroxynitrite in the lower nanomolar range. Furthermore, the in vivo experiments in GF mice were short-term (2 h), which may make it difficult to draw major conclusions for a chronic setting.

## 5. Conclusions

Taking together all results, inorganic nitrite represents a potent vasodilator, which is not significantly impaired by ex vivo oxidative stress. This may be secondary to the oxidative conversion of XDH to XO, which has been proposed as a mechanism for nitrite bioactivation. In addition, the administration of nitrite leads to an increased formation of S-nitrosocysteine-positive proteins, and the gastrointestinal flora mediates these effects as proven by our studies in GF mice. However, acute administration of nitrite does not beneficially influence the endothelial function in conventionally-raised mice. This may offer attractive therapeutic options for the use of probiotics in the intervention of hypertension, as discussed above. In conclusion, the concept that nitrite bioactivation is promoted in areas or vascular beds with evident oxidative stress is highly attractive and may explain the beneficial profile of nitrite therapy in various ischemic heart diseases. The redox conversion of XDH to XO obviously generates a major enzyme for nitrite bioactivation, supporting the persistent vasodilatory action of nitrite under oxidative stress conditions. As a future perspective, studies in XDH knockout-mice may be conducted to reveal the importance of XDH to XO conversion for the protective effects of inorganic nitrite in mice with cardiovascular disease.

## Data Availability

All study data are presented in the main text. Raw data and experimental materials used in this study are available upon reasonable request to the corresponding author.
